# Intensive interventions for emotionally based school avoidance: a scoping review of program structures and therapeutic levers

**DOI:** 10.3389/fpsyt.2026.1851117

**Published:** 2026-07-31

**Authors:** Lux Ratnamohan, Erin Smith, Peter McInnis, Rebecca Somervaille, Sophie Champion, Hugh Cooney, David Heyne

**Affiliations:** 1 Rivendell Child & Adolescent Mental Health Service, Thomas Walker Hospital, Sydney Local Health District, Sydney, NSW, Australia; 2 School of Medicine, University of Sydney, Sydney, NSW, Australia; 3 School of Psychology, University of Sydney, Sydney, NSW, Australia; 4 Canterbury Adolescent Outreach Service, Sydney Local Health District, Sydney, NSW, Australia; 5 School of Psychology, Deakin University, Geelong, VIC, Australia

**Keywords:** emotionally based school avoidance (EBSA), emotion-related school attendance challenges, intensive intervention, multi-tiered system of supports (MTSS), school attendance problems, school refusal

## Abstract

**Background:**

A substantial proportion of children and adolescents with emotionally based school avoidance (EBSA) do not benefit from outpatient mental health interventions. In such instances, more intensive interventions may be warranted. There is a lack of synthesised knowledge, however, to guide professionals and families about what intensive interventions comprise, how they generate change, and when they are indicated.

**Objective:**

To map and synthesise the literature on intensive interventions for EBSA, in terms of target populations, intervention structure and delivery, therapeutic levers, implementation challenges, and reporting completeness.

**Methods:**

A systematic search of five databases and grey literature sources (2000–2026) identified 1,581 records. Following screening and full-text review, 19 publications describing 17 samples across 13 programs met inclusion criteria. Data were charted using a structured coding manual informed by TIDieR, the Medical Research Council framework for complex interventions, and STROBE reporting guidelines. Numerical summaries were combined with narrative synthesis to describe patterns in sample characteristics, intervention structure, therapeutic levers, implementation challenges, and reporting completeness.

**Findings:**

Intensive interventions commonly targeted adolescents with severe, often chronic, school non-attendance and substantial psychiatric comorbidity. Four program types were identified: outpatient, alternative education, day hospital, and inpatient. The latter three types utilised a transitional setting staffed by a multidisciplinary team to deliver integrated health and education support. High-intensity graded exposure, educational scaffolding, therapeutic peer milieu, health-education systems coordination, and the transitional space were key therapeutic levers for facilitating engagement and eventual return to school. Common implementation challenges included sustaining engagement, managing progression through treatment, and resource constraints. Whilst the reported outcomes were promising, inconsistent reporting limited inferences about the indications for intensive interventions.

**Conclusions:**

Intensive EBSA interventions are best understood as programs that restructure the conditions of care to enable implementation of evidence-based treatments. This review identified a typology of intensive intervention models and recurring therapeutic levers, providing a foundation for future research. Improved specification of the target population and intervention models, together with investigation of the mechanisms of change, will help clarify their role within multi-tiered intervention frameworks for EBSA.

## 1 Background

Emotionally Based School Avoidance (EBSA), also known as School Refusal, is a complex, transdiagnostic syndrome characterised by a young person’s non-attendance due to emotional distress despite reasonable efforts by family to promote attendance (1). When severe, EBSA disrupts psychosocial development and jeopardises educational and vocational opportunities, as well as imposing a substantial burden on families. Severe forms of EBSA disproportionately affect young people with psychiatric disorders, making it a potent pathway along which disadvantage accrues (2).

Rates of EBSA appear to have risen dramatically in the past decade, a trend predating but potentiated by the COVID-19 pandemic (3), and mirroring the broader rise in youth mental health problems (4). With the rise in EBSA has come growing recognition of the need for multi-tiered intervention frameworks, coordinated across the sectors of education and health. In multi-tiered intervention frameworks, resource allocation is stratified to extend the reach of interventions to the whole-of-school population, whilst also directing the most intensive interventions to those with the most severe problems (5). One framework proposed is Kearney and Graczyk's (2014) Multi-Tiered System of Supports (MTSS). In this model, Tier 1 comprises universal, preventative measures delivered to the whole-of-school population to promote a positive school climate and foster school connectedness. Tier 2 comprises targeted interventions, delivered by school staff to provide additional support to ‘at-risk’ students with mild or emerging EBSA. Tier 3 comprises ‘expanded’ interventions delivered by mental health clinicians working collaboratively with school personnel to support students with persistent EBSA (6). Common examples of Tier 3 interventions include weekly outpatient cognitive behavioural therapy (7) and multi-modal therapy (8).

A key finding from intervention research on EBSA is the high proportion of youth who do not benefit from Tier 3 interventions. Several randomised controlled trials of Tier 3 interventions demonstrate that between one to two-thirds of participants will not achieve a satisfactory return to school (9, 10). Moreover, response rates reported from RCT data may over-estimate those seen in real-world practice. For example, a Swedish observational study of a long-running treatment program for EBSA found that less than a fifth of adolescents successfully returned to school after a year of weekly clinic-based CBT (11).

When a young person does not respond to evidence-based, Tier 3 clinical interventions, more intensive forms of support may be warranted. Intensive interventions for EBSA are hardly novel (12, 13), yet the literature describing such interventions remains fragmented, reflecting several challenges specific to the field. Intensive EBSA interventions are complex, typically comprising interacting components delivered across multiple systems. Furthermore, they are often context-dependent, reflecting local educational and health service policy, configuration, and resourcing. In addition, the populations receiving intensive interventions are heterogeneous, encompassing young people with varying patterns of attendance difficulties, psychiatric and neurodevelopmental profiles, and prior treatment histories. These features limit the feasibility of evaluation using controlled trial designs, leaving the field reliant primarily on observational studies, case series, and program descriptions. Finally, until recently, intensive EBSA interventions have not been part of a unified conceptual field, instead being described within separate traditions of education and mental health research. As a result, stakeholders lack integrated evidence to guide decision making for young people with complex and severe forms of EBSA.

The recent emergence of multi-tiered frameworks provides an opportunity to conceptualise intensive interventions as part of a broader continuum of support, rather than as isolated service models. In Kearney’s framework, for example, Tier 4 interventions are described as “innovative, creative, intensive and typically nonbureaucratic approaches” in the form of “specialized programs”, for students with “such severe and chronic attendance problems that even extensive Tier 3 efforts may be inadequate or insufficient” (6, p. 114-115). Such frameworks provide a useful conceptual lens for considering the role of intensive interventions within a broader continuum of care. Addressing the role of intensive EBSA interventions within multi-tiered frameworks, however, first requires a clearer understanding of who these interventions are intended for, what they consist of, and how they are intended to generate change. This, in turn, requires sufficient detail regarding participant selection, intervention design, and outcomes to determine the applicability of findings across settings.

For fields early in their development, scoping review methodologies offer a means of reconnaissance, in which researchers conduct a “preliminary assessment of potential size and scope of research literature” (14, p. 95), in order to “envisage where gaps and innovative approaches may lie” (15, p. 28). Applied to intensive EBSA interventions, this approach enables systematic mapping of the populations for whom interventions are used, the structure and delivery of interventions, the therapeutic levers through which they are intended to generate change, the implementation challenges encountered during delivery, and the completeness of reporting across studies. This, in turn, enables identification of evidence gaps, informs future research priorities, and provides a foundation for considering the role of intensive interventions within multi-tiered frameworks.

*Objectives*

We sought to:

- Clarify the populations for whom intensive EBSA interventions are used, in terms of referral pathways, eligibility criteria, and sample characteristics;
- Map the structure and delivery of intensive EBSA interventions in terms of program setting, staffing, intensity, duration, treatment components, and reintegration pathways;
- Identify the therapeutic levers through which these interventions are intended to generate change;
- Identify the implementation challenges encountered in the delivery of intensive EBSA interventions; and
- Assess the completeness with which participant characteristics, intervention characteristics, and outcomes are reported across studies.

For consistency, we use the term EBSA to denote school refusal and related forms of emotionally driven non-attendance. Original study terminology (e.g., school refusal, absenteeism, school avoidance) is retained where necessary to preserve the language of the source studies.

## 2 Method

We conducted this scoping review in accordance with PRISMA-ScR (16).

### 2.1 Eligibility criteria

Eligibility criteria were developed using the Population–Concept–Context (PCC) framework recommended for scoping reviews. The population comprised school-aged children and adolescents experiencing EBSA; the concept was intensive EBSA interventions; and the context included educational and mental health settings. Studies were eligible for inclusion if they met all the following criteria (1): Year: Published between January 2000 and February 2026 inclusive (2); Type: published in peer-reviewed journal article, or university dissertation (3); Accessibility: Full text accessible (4); Population: School-age children or adolescents experiencing EBSA (5); Design: Empirical report employing quantitative, qualitative, mixed methods, case reports, or program description (6); Intervention: Met all of the following criteria: (i) High intensity: Treatment delivered ≥3 sessions per week for the majority of intervention period; (ii) Primary focus: Intervention designed to target EBSA as a primary treatment goal; (iii): Setting: Treatment organised and delivered primarily by staff external to the young person’s enrolled school (hereafter referred to as the “home school”); (iv) Time limited: Defined duration, with the aim of facilitating re-entry into longer-term educational or vocational pathway (7); Minimum reporting threshold: Studies had to provide sufficient information to characterise the intervention in terms of setting, structure, and treatment components.

This timeframe was selected to capture interventions developed within contemporary mental-health and educational contexts. No language restrictions were applied and a broad range of study types and sources (e.g., university dissertations) were included to reflect the exploratory aims of the review. Full-text accessibility and minimum reporting thresholds were required to permit extraction and characterisation of intervention structure and delivery. Intervention criteria were designed to identify programs providing intensive, time-limited treatment for EBSA as a primary treatment target, whilst excluding standard-intensity outpatient interventions, transdiagnostic programs not specifically designed to address EBSA, and interventions delivered primarily within the young person’s home school.

### 2.2 Data sources

Initially, we systematically searched PsycINFO, MEDLINE, Embase, ERIC, and ProQuest Dissertations & Theses Global for peer-reviewed articles and dissertations published between January 2000–August 2025, with no language limits. Search terms were developed by a researcher with expertise in EBSA [DH]. The database search yielded 1,632 records; 1,539 remained after de-duplication. To address cross-disciplinary indexing limitations, we used additional search strategies: (i) targeted Google Scholar queries using simplified core phrases (e.g., “school refusal,” “school avoidance,” “day program,” “partial hospital,” “alternative education program,” “intensive”), screening the first 100 results for each query; (ii) hand-searching key journals identified in a recent bibliometric analysis (17); and (iii) contact with authors and field experts to locate additional published or unpublished work. These strategies yielded an additional 42 records for screening. To ensure currency of the review, an updated search was conducted to identify studies published between August 2025 and February 2026, yielding an additional two records.

### 2.3 Screening

A two-stage screening process was applied to the 1,583 records identified. Records were managed in EndNote for de-duplication and imported into Covidence for screening. At stage one, titles and abstracts were screened against the eligibility criteria. Each record was independently reviewed by two researchers, with disagreements referred to a third reviewer (PM). Following this stage, 69 records were retained for full-text review. At stage two, full texts were again assessed independently by two researchers. Screening of non-English texts (n = 9) was conducted using Google Translate, from which 5 publications were ultimately included. Disagreements occurred for 10 publications (14.5%) and were resolved through consensus with the third reviewer. Nineteen publications describing 17 samples across 13 programs were ultimately included for data extraction.

### 2.4 Data extraction

Data charting was iterative (18). Because no single framework captured the clinical, educational, and service-delivery dimensions of intensive EBSA interventions, the extraction guide integrated elements from several established frameworks. The Medical Research Council Framework for Developing and Evaluating Complex Interventions (MRC Framework) informed extraction of intervention theory and program logic (19). The Template for Intervention Description and Replication (TiDieR) was used to extract data on intervention structure and components (20). The Strengthening the Reporting of Observational Studies in Epidemiology (STROBE) framework was used to extract data on study design, sample characteristics and context (21).

In the first round we drafted a preliminary extraction template based on prior knowledge of the literature and piloted it by having two team members independently extract data from five of the included studies. Following team discussion, the template was refined to improve feasibility and coverage. In the second round, two team members independently charted data for all included studies, with discrepancies resolved by team discussion. Based on emergent patterns and themes, a structured codebook was then developed covering the domains of intervention setting, structure, modalities, reintegration processes, theoretical foundations, program logic, and implementation challenges. In the third round, two extractors independently applied codes and descriptive themes for each domain. Disagreements were resolved by discussion and, where consensus could not be reached, by third-reviewer adjudication. Data extracted and coded across these domains were subsequently synthesised and summarised in three tables: a comparative matrix of study characteristics (**Table 1**), a narrative matrix of intervention characteristics (**Table 2**), and a reporting completeness grid (**Table 3**; see **Supplementary Appendix**).

**Table 1:** Study characteristics of included publications (n = 19; 17 samples).

Sample ID	Author & year	Aim	Design	Eligibility criteria	Referral pathway	Sample size & demographics	EBSA duration	Psychiatric morbidity	Key findings
01	Alais 2023 (22)	To describe the role of a specialist inpatient care-and-education unit in treating of an adolescent with anxious school refusal and internet gaming disorder.	Case report	Age 15 – 25 + MH / addiction problems w/ severe educational impact	NR	N=1 15 y.o male	12 months	Behavioural disorder (internet gaming disorder)	Following inpatient hospitalisation, this case showed improved functioning and progressive re-engagement with education.
02	Brouwer-Borghuis 2017 (23)	How does a specialised alternative educational program for school refusal operate, what are the characteristics of adolescents referred to it, and what educational re-engagement outcomes are observed?	Retrospective cohort	Age 12 – 19 + meet SR criteria	School	N = 30 ages 12–17 40% male	Mean = 6 months	Autism spectrum disorder predominant (with comorbid internalising disorders)	Most adolescents re-engaged with education following the program, with 79% transitioning to special education settings.
03	Chu 2015 (24)	Can DBT be adapted into a multimodal programme (DBT-SR), including web-based coaching, to target mechanisms maintaining school refusal?	Prospective cohort (single arm, pre–post)	Age 12 – 16 + meet SR criteria	Open	N = 4 ages 13–16 75% male	NR	Mixed internalising disorders	In this pilot DBT-SR trial, 2/4 participants completed treatment; among completers, attendance and diagnostic status improved, but engagement was limited by early dropout and inconsistent participation.
	Zendegui 2015 (25)	In an open trial of DBT-SR, is web-based coaching (WBC) feasible and acceptable to young people, parents, and therapists, and what functions does it serve?	Mixed-methods study (feasibility)						In a DBT-SR pilot trial, parents and therapists rated web-based coaching as feasible and acceptable, whereas youth ratings were lower; qualitative feedback suggested support for real-time problem-solving and skills generalisation, but retention was limited.
04	Denis 2018 (26)	Does a day-hospital CBT program improve school reintegration, anxiety symptoms, and functioning in adolescents with anxious school refusal?	Retrospective cohort	Age 11 – 16 + meet SR criteria + anxiety disorder	NR	N = 28 ages 11–16 35% male	NR	Anxiety disorders	Following a day-hospital CBT program, 96% returned to full-time school; functioning and anxiety symptoms improved from pre- to post-treatment.
05	Denis 2026 (27)	Does an intensive day-hospital CBT program achieve clinically meaningful school attendance (≥80%) and improve anxiety and functioning in adolescents with severe anxious school refusal?	Prospective cohort (single arm, pre–post)	Age 11 – 16 + meet SR criteria + anxiety disorder	NR	N = 65 ages 10–16 30% male	Median = 2 months	Anxiety disorders	Following a day-hospital CBT program, 53% achieved ≥80% attendance at 6 months; improvements in anxiety and functioning were maintained at 12 months.
06	Grandison 2011 (28)	How do young people and parents experience reintegration from a Short Stay School to mainstream education following school refusal?	Qualitative study (case series)	NR	Education authority alternative provision placement	N = 5 ages 11–16 60% male	NR	NR	Reintegration from the Short Stay School to mainstream was emotionally challenging for families. Facilitators included personalised and phased planning, a clear focus on return from the outset, identification of a key worker, parent–school collaboration, and relational continuity. Barriers included parental anxiety, bullying concerns, inflexible school responses, poor communication, and young person resistance to reintegration.
07	Hannan 2019 (29)	Does intensive (daily) CBT for school refusal improve school attendance (≥90%) and related clinical outcomes in young people with school refusal associated with anxiety/depression?	Prospective cohort (single arm, pre–post)	Met SR criteria + anxiety / depressive disorder	Outpatient MH clinic	N = 25 ages 9–18 52% male	NR	Mixed internalising disorders	Following intensive daily CBT, 60% achieved ≥90% attendance post-treatment; clinician-rated global severity and self-reported depressive but not anxiety symptoms improved. Four of 25 participants discontinued treatment.
08	Hilwerling 2020 (30)	What factors are associated with successful treatment of chronic school absenteeism in a specialised psychiatric day-unit program?	Retrospective cohort	Chronic absenteeism + psychiatric disorder	Outpatient MH clinic	N = 67 ages 7–17 gender NR	Range = 3–24 months	Mixed internalising and behavioural disorders.	Following the hospital day program, 82% (55/67) achieved regular school attendance at 6-month follow-up.
09	Iwamitsu 2007 (31)	In adolescents with school refusal and delayed sleep phase syndrome (DSPS), does hospitalisation improve sleep–wake rhythm and school attendance?	Prospective cohort (single arm, pre–post)	Met SR criteria + delayed sleep phase syndrome	NR	N = 22 ages 13–18 gender NR	NR	Delayed sleep phase syndrome	Following inpatient hospitalisation, sleep–wake rhythm improved in almost all participants, and 57% maintained improvement after discharge with associated attendance gains (>70% attendance). Poorer outcomes were associated with low assertiveness and emotional suppression.
10	McKay-Brown 2019 (32)	In adolescents with school refusal, does a multidisciplinary home–school–clinic intervention (In2School) improve school re-engagement and attendance, mental health, functioning and quality of life?	Prospective cohort (single arm, pre–post)	Age 11 – 15 + met SR criteria + DSM-5 diagnosis	Tertiary MH service	N = 7 ages 12–14 43% male	Mean = 13 months	Mixed internalising disorders	Following the In2School day program, 7/7 participants achieved successful return to school, and 5/7 had maintained ≥70% attendance at 6-month follow-up, with associated improvements in functioning and quality of life.
11	Neumann 2022 (33)	How is schema therapy implemented in an inpatient setting for adolescents with chronic school avoidance, and what changes are observed in a case example?	Case report	NR	NR	N = 1 16 y.o. male	18 months	Depressive disorders	Following inpatient treatment, this case showed improved functioning and successful reintegration to school.
12	Oner 2014 (34)	How can a multimodal inpatient treatment, including staged CBT-based exposure, be applied to severe treatment-resistant school refusal, and what outcomes are observed in two cases?	Case series	NR	NR	N = 2 ages 8–15 50% male	NR	Anxiety disorders	Following inpatient treatment, 2/2 cases achieved school re-entry and maintained attendance at 6–8 month follow-up.
13	Sibeoni 2018 (35)	How do adolescents and their parents experience psychiatric care for SR, and what do they perceive as the key “active ingredients” of care?	Qualitative study (inductive thematic analysis)	NR	NR	N = 20 ages 12–18 gender NR	NR	Anxiety disorders	Adolescents and parents described intensive psychiatric care as providing time, containment and therapeutic relationships that supported gradual change. Adolescents emphasised internal recovery (confidence, symptom relief, autonomy), whereas parents prioritised school reintegration and restoration of routine.
14	Tolin 2009 (36)	Does intensive behaviour therapy improve outcomes in adolescents with severe school refusal?	Single-case experimental design (multiple baseline)	Met SR criteria + anxiety / depressive disorder	Local school network	N = 4 ages 13–16 100% male	Range = 3–12 months	Mixed internalising disorders.	Following intensive outpatient CBT, 3/4 adolescents returned to full-day attendance by discharge; at 3-year follow-up, all available cases were rated by parents as improved, although educational pathways varied.
15	Walter 2009 (37)	How did an inpatient and step-down CBT-oriented programme support school reintegration for an adolescent with emotionally driven school refusal?	Case report	NR	NR	N = 1 14 y.o M	6 months	Mixed internalising disorders	Following inpatient treatment, this case showed improved functioning and successful graded return to school.
16	Walter 2010 (38)	What are the short-term changes in school attendance and mental health symptoms following inpatient treatment (including CBT) for anxious-depressed adolescents with severe school absenteeism?	Prospective cohort (single arm, pre–post)	Age 12 – 18 + school absenteeism + psychiatric diagnosis	Outpatient mental health clinics	N = 147 ages 12–18 57% male	Mean = 5 months	Mixed internalising disorders	Following inpatient treatment, continuous attendance increased to 87.1% at discharge and 82.3% at 2-month follow-up; mental health symptoms also improved.
	Walter 2013 (39)	Which baseline demographic/clinical/psychosocial factors predict school attendance and symptom outcomes after inpatient CBT for anxious-depressed school absenteeism?	Predictor analysis (cohort study)						In predictor analyses of the treated cohort, no baseline variables predicted attendance or symptom outcomes.
17	Walter 2014 (40)	Do attendance and symptom changes occur specifically during treatment (vs a short untreated waiting period), and are effects maintained at follow-up?	Prospective single-arm design – cohort (pre–post with short pre-treatment waiting period comparison)	Age 12 – 18 + school absenteeism + psychiatric diagnosis	Outpatient mental health clinics	N = 36 ages 12–18 58% male	Mean = 5 months	Mixed internalising disorders	Following inpatient treatment, continuous attendance increased to 88.9% at discharge and 63.9% at 9-month follow-up; symptom change was not statistically significant.

This table summarises study-level characteristics for each included publication. Where multiple publications reported on the same sample (e.g., Walter 2010 and 2013; Chu 2015 and Zendegui 2015), split rows are used. The Aim column reflects terminology from the original publication where possible (e.g. school refusal, school absenteeism). The Eligibility Criteria column presents simplified inclusion characteristics. Specific non-attendance criteria were not reproduced, as clinically significant school non-attendance was a defining feature across all included studies and showed limited meaningful variation. The Referral Pathway column indicates the primary source of referral into the intervention. The Sample Size & Demographics column reports sample size (N), age range, and gender distribution. The EBSA Duration column summarises the duration of school non-attendance prior to intervention, as reported in the study (mean, median, or range where available). The Psychiatric Morbidity column provides a standardised summary of the clinical profile of each sample, using broad diagnostic groupings (e.g. anxiety disorders, mixed internalising disorders), based on author-reported diagnoses. The Key Findings column summarises the primary outcomes of each study.

SR, school refusal; CBT, cognitive behavioural therapy; DBT, dialectical behaviour therapy; MH, mental health; NR, not reported.

**Table 2:** Programme characteristics of intensive EBSA interventions (n = 13 programs).

Author & year | city / region, country	Program type & site	Theoretical orientation	Staffing	Weekly intensity (Sessions / Hrs)	Duration (wks)	Psychotherapy (recipients & modalities)	Non-psychotherapy modalities	Re-integration pathway	Re-integration structure
Alais 2023 (22) Sceaux, IDF, FRA	Inpatient Hospital	Systemic	MH + EDU	6d/wk	24*	Fam (MDFT) Par.Ind (MDFT) Ind (NR) Grp (Psychoed)	Transitional Classroom Case Management Creative Arts Therapies Pharmacotherapy	Indirect	Graded
Brouwer-Borghuis 2017 Almelo, NL	Day Program School	Ecological	EDU + MH	5 days / wk	50	NIL	Transitional Classroom Case Management	Indirect	Graded
Chu 2015, Zendugui 2017 (24) New Brunswick, NJ, USA	Outpatient Home / Clinic	DBT	MH	2 sessions / wk ~ 4hrs / wk + daily web-based coaching	16	Ind (DBT-SR) Par (Coaching) Fam (NR) Fam.Grp (MFT/DBT-skills)	Case Management	Direct	Graded
Denis 2018, 2026 (26, 27) Montpellier / Marseille / Beziers / Nimes, FR	Day Program Hospital	CBT	MH + EDU	4 days / wk	19 - 30	Ind (CBT) Grp (CBT) Par (Psychoed) Par.Grp (Psychoed)	Transitional Classroom Case Management	Indirect	Graded
Grandison 2011 (28) Birmingham, UK	Day Program School	Ecological	EDU	5 days / wk	6**	NIL	Transitional Classroom Case Management	Indirect	Graded
Hilwerling 2020 (30) Munster, GER	Day Program Hospital	Behavioural	MH + EDU	5 days / wk	15	Ind (BT) Grp (BT) Par (NR) Fam (NR) Fam.Grp (MFT)	Transitional Classroom Case Management Creative Arts Therapies Transport	Indirect	Graded
Iwamitsu 2007 (31) Shiga, JP	Inpatient Hospital	Behavioural	MH	7 days / wk	NR	Grp (NR)	Sleep-Wake Modification Medical Consultation	Direct	NR
McKay-Brown 2019 (32) Melbourne, AUS	Day Program School	Ecological	EDU + MH	4 days / wk ~ 23 hours / wk	14	Ind (CBT) Grp (CBT-skills) Par (BPT) Par.Grp (BPT)	Transitional Classroom Case Management Medical Consultation	Indirect	Graded
Neumann 2022 (33) Essen, GER	Inpatient Hospital	Schema	MH + EDU	5d / wk	12	Ind (Schema) Grp (Schema / Skills) Fam (Schema)	Transitional Classroom Case Management Medical Consultation	Indirect	Graded
Oner 2014 (34) Ankara, TUR	Inpatient Hospital	Behavioural	MH	7d / wk	3 – 5*	Ind (CBT) Grp (CBT-Skills) Par (Psychoed) Fam (NR)	Case Management Medical Consultation Pharmacotherapy	Direct	Graded
Sibeoni 2018 (35) Argenteuil / Rouen / Marseille, FR	Inpatient Hospital	Systemic	MH + EDU	NR	NR	Ind (CBT / Dynamic / IPT) Grp (NR) Fam (Systemic)	Transitional Classroom Creative Arts Therapies Pharmacotherapy Case Management	Indirect	NR
Tolin 2009 (36) Hannan 2019 (29) New Haven, USA	Outpatient Clinic	CBT (Functional Analytic model of School Refusal Behaviour)	MH	5 sessions / wk ~ 10 hrs / wk	3**	Ind (CBT) Par (BPT) Fam (NR)	Case Management	Direct	Graded
Walter 2008, 2010, 2014, 2013 (38–40) Cologne, GER	Inpatient Hospital	CBT	MH + EDU	5d/ wk	8	Ind (CBT) Grp (CBT) Par (BPT / psychoed) Fam (NR)	Transitional Classroom Pharmacotherapy Case Management	Flexible	Graded

This table summarises program-level characteristics of included interventions. Where multiple publications described the same program, data are combined into a single row. Program Type & Site classifies interventions by primary delivery structure (outpatient, day programme, inpatient) and setting (school, hospital, clinic). Theoretical Orientation describes the reported or inferred theoretical foundation of the program. Where multiple influences were present, the primary organising model was selected. Staffing indicates the composition of the delivery team, coded by sector involvement. The sector with greater representation is listed first. Weekly Intensity reports program frequency, either as days, sessions, or hours per week. Duration reflects programme length in weeks (mean, median, range, or case duration, as reported). Asterisks denote values derived from case-level data (*), or from intended programme durations (**). Psychotherapy modalities are coded in the format of recipient (modality). Recipient codes are Ind, individual; Grp, group; Par, parent; Fam, family. Modality codes are CBT, cognitive behavioural therapy; DBT, dialectical behaviour therapy; BT, behavioural therapy; BPT, behavioural parent training; IPT, interpersonal therapy; MDFT, multidimensional family therapy; MFT, multi-family therapy. (NR) indicates psychotherapy was delivered but modality was not specified; “NIL” indicates no formal psychotherapy component was described. Non-psychotherapy modalities capture additional programme components. Reintegration pathway describes the route back to school: direct (return to home school), indirect (attendance first to program site classroom, followed by reintegration to home school), or flexible (both pathways used). Reintegration structure indicates whether reintegration followed a graded (stepwise) process or was not explicitly structured.

CBT, cognitive behavioural therapy; DBT, dialectical behaviour therapy; BT, behavioural therapy; BPT, behavioural parent training; IPT, interpersonal therapy; MDFT, multidimensional family therapy; MFT, multi-family therapy; MH, mental health; EDU, education; NR, not reported; NIL, not described.

**Table 3:** Reporting characteristics of included EBSA intervention studies.

Author & year	EBSA severity	Program logic	Intervention description	Selection, engagement & attrition	Outcome domains	Longitudinal f/u
Alais 2023 (22)	Attendance rate Comorbidity Treatment History	Explicit	Complete	Engagement	Attendance	NR
Brouwer-Borghuis 2017	Attendance rate Chronicity Comorbidity	Explicit	Complete	NR	Educational setting	NR
Chu 2015, Zendugui 2017 (24)	Attendance rate Comorbidity	Explicit	Complete	Engagement Attrition	Attendance Symptomology Global Fn	4 months
Denis 2018 (26)	Attendance rate Chronicity Comorbidity	Implicit	Complete	Engagement Attrition	Attendance Symptomology Global Fn	NR
Denis 2026 (27)	Attendance rate Chronicity Comorbidity	Implicit	Complete	Selection Engagement Attrition	Attendance Symptomology Global Fn	12 months
Grandison 2011 (28)	NR	Explicit	Partial	NR	NR	NR
Hannan 2019 (29)	Attendance rate Comorbidity	Explicit	Complete	Selection Attrition Engagement	Attendance Symptomology Global Fn SR behaviour	3 months
Hilwerling 2020 (30)	Attendance rate Chronicity Comorbidity Treatment History	Explicit	Complete	Attrition	Attendance	6 months
Iwamitsu 2007 (31)	Attendance rate	Explicit	Partial	NR	Attendance Symptomology	3 months
McKay-Brown 2019 (32)	Attendance rate Chronicity Comorbidity	Explicit	Complete	Selection Attrition Engagement	Attendance symptomology Global Fn QoL	6 months
Neumann 2022 (33)	Attendance rate Chronicity Treatment history Comorbidity	Explicit	Complete	Engagement	Attendance Symptomology	NR
Oner 2014 (34)	Attendance rate Chronicity Treatment hx Comorbidity	Explicit	Complete	Engagement	Attendance Symptomology	6 – 8 months
Sibeoni 2018 (35)	Comorbidity	NR	NR	Engagement	NR	NR
Tolin 2009 (36)	Attendance rate Chronicity Comorbidity	Implicit	Complete	Selection Attrition Engagement	Attendance Symptomology Global Fn	3 years
Walter 2008	Attendance rate Chronicity Comorbidity Treatment History	Explicit	Complete	Engagement	Attendance Symptomology	6 months
Walter 2010, 2013 (38) (39)	Attendance rate Chronicity Comorbidity Treatment History	Implicit	Partial	Selection (2014) Attrition	Attendance Symptomology Global Fn	9 months
Walter 2014 (40)	Attendance rate Chronicity Comorbidity Treatment History					9 months

This table summarises the reporting of key domains across included studies. Where multiple publications described the same study sample, data is combined at the sample level. Coding reflects what was reported for each sample, not the quality of reporting. Reporting of EBSA Severity indicates which domains were explicitly reported prior to intervention entry (e.g. attendance rate, chronicity, comorbidity, treatment history). Program logic reflects whether an explicit rationale linking intervention components to mechanisms or targets was described (Explicit), implied through theoretical orientation without mapping (Implicit), or not reported (NR). Intervention description indicates whether programme components, structure, and delivery were described with sufficient detail to allow meaningful comparison across studies (Complete, Partial, NR). Reporting of Selection, Engagement & Attrition indicates which domains were explicitly reported, either quantitatively or qualitatively. Outcome domains lists the domains assessed or explored, including attendance, symptomology, global functioning, and related constructs. Longitudinal f/u indicates whether outcomes were assessed beyond discharge, reported as the duration of follow-up where available. Only domains explicitly reported in the study are listed; absence of a domain indicates it was not reported.

NR, not reported; Fn, functioning; QoL, quality of life; SR, school refusal.

### 2.5 Data synthesis

Data synthesis followed a combination of descriptive numerical summary and structured narrative synthesis, consistent with scoping review methodology (16, 18, 41). Descriptive synthesis summarised study, sample and program characteristics using frequency counts (e.g., country, setting, duration, modality, theoretical orientation, and outcome domains) to summarise the scope and distribution of the evidence base. As some samples and interventions were reported across multiple publications, different units of analysis were used throughout the review. Publication characteristics are reported at the publication level (n = 19), participant characteristics at the sample level (n = 17 unique samples), and intervention characteristics at the program level (n = 13 programs). For clarity, all interventions are referred to as “programs” throughout the Results section.

Data synthesis proceeded at two levels. First, programs were grouped according to their primary delivery structure, resulting in four program *types*: intensive outpatient therapy, alternative education programs, day hospital programs, and inpatient programs. Within each program type, narrative synthesis was used to summarise structural features, positioning within health and education systems, and reported outcomes.

Second, a cross-type analysis examined recurring processes and implementation challenges operating across program types. Therapeutic levers and implementation challenges were derived using a common inductive and comparative approach. Therapeutic levers were defined as processes or programme features that appeared to facilitate engagement, participation, or change. Candidate therapeutic levers and implementation challenges were identified from coded data on program components, theoretical foundations, program logic, and descriptive accounts. Cross-program comparison was then used to consolidate recurring therapeutic levers and implementation challenges into higher-order categories that were representative of the body of reviewed studies.

Consistent with scoping review methodology, no formal risk-of-bias appraisal was conducted.

## 3 Results

Results are presented in five sections: selection of sources of evidence (3.1); characteristics of included studies and programs, including reporting completeness (3.2); synthesis of intervention types (3.3); and synthesis of therapeutic levers (3.4), and implementation dilemmas (3.5).

### 3.1 Selection of sources of evidence

**Figure 1:**
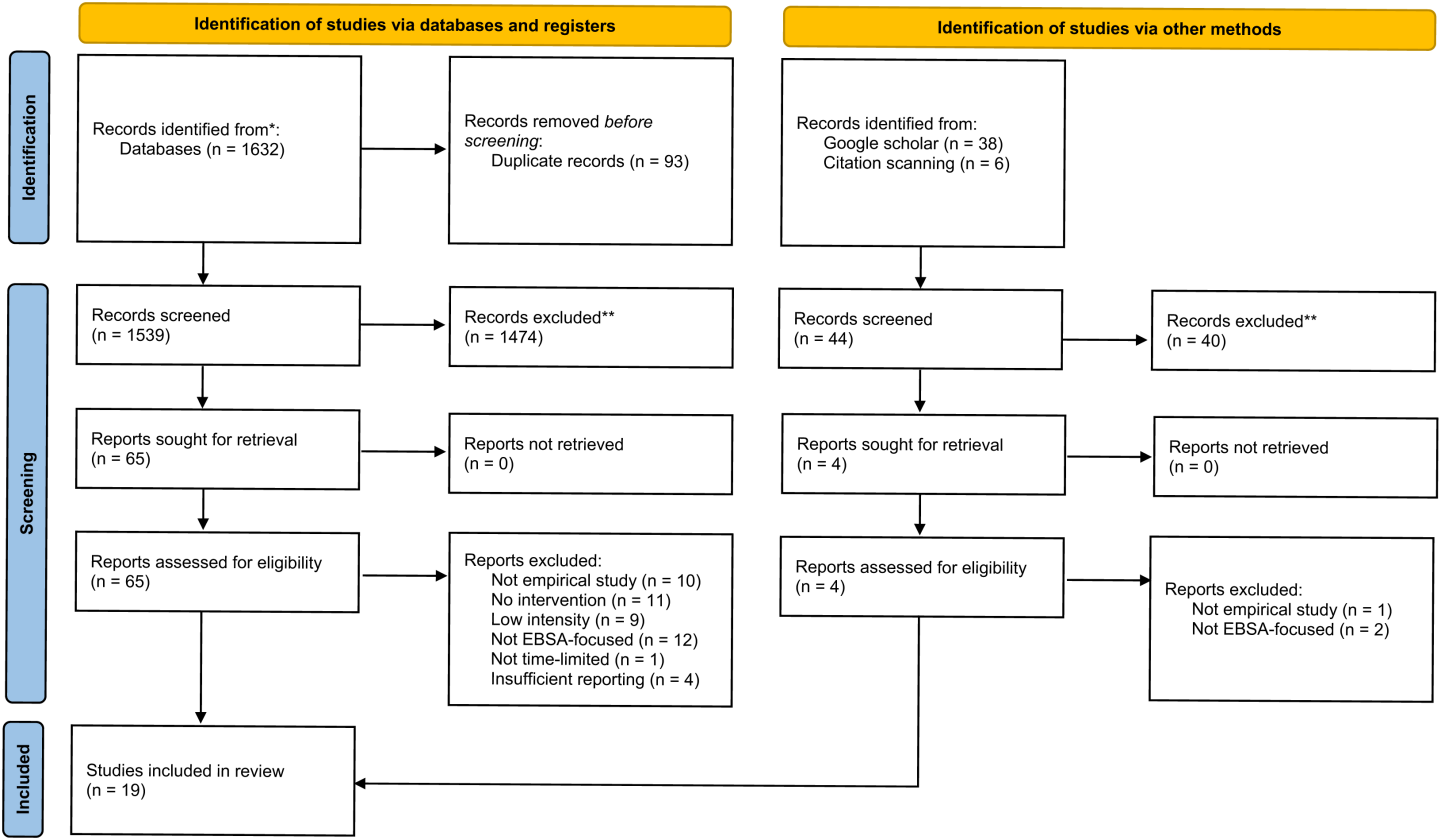
PRISMA 2020 flow diagram of study selection. This figure depicts the process of study identification, screening, eligibility assessment, and inclusion. Database searches yielded 1,632 records, with 1,539 remaining after de-duplication. Additional records were identified through other methods, including Google Scholar (n = 38) and citation scanning (n = 6). Following title and abstract screening, 69 reports were retained for full-text review. Of these, 50 reports were excluded for reasons including non-empirical design, absence of an intervention, low-intensity interventions, lack of focus on school refusal/emotionally based school avoidance (EBSA), non–time-limited interventions, or insufficient reporting. Nineteen publications describing 13 distinct programs were included in the review.

In total, 1,582 records were screened and 69 retained for full-text review. Following full-text review, 19 publications describing 17 samples across 13 programs met inclusion criteria. Reasons for exclusion at the full-text stage included non-empirical study design (n = 11), absence of an eligible intervention (n = 11), insufficient intervention intensity (n = 9), lack of focus on EBSA (n = 13), non-time-limited intervention (n = 1), and insufficient intervention detail to permit data extraction (n = 4). The study selection process is summarised in **Figure 1** (PRISMA flow diagram).

### 3.2 Characteristics of included studies and programs

#### 3.2.1 Study characteristics

Of the 19 publications, 17 were peer-reviewed journal articles and two were university dissertations. Publications spanned 2007–2026, with almost half published since 2017. Interventions were delivered in eight countries across four continents, most commonly France (n = 3), Germany (n = 3), and the USA (n = 2). As outlined in **Table 1**, studies sought to describe an intervention (n = 9), evaluate its effectiveness (n = 8), explore stakeholder perspectives (n = 2), establish intervention feasibility (n = 1) and/or identify outcome predictors (n = 1). Effectiveness studies used prospective single-arm designs (n = 7) or retrospective cohort analysis (n = 1). Descriptive studies used retrospective cohort designs (n = 5) and case reports (n = 4). Stakeholder perspectives were explored using thematic analysis (n = 1) and interpretive phenomenology within a case-study framework (n = 1).

Sample sizes ranged from single-case reports to large cohorts (N = 1-147, median 14). Most samples comprised adolescents with a similar representation of males and females. The duration of school attendance problems ranged from 3–24 months. Psychiatric morbidity was high across samples, with anxiety disorders being the most frequently reported diagnoses followed by depressive disorders. Amongst studies reporting on the presence of psychiatric comorbidity, between 37-88% of participants had multiple psychiatric diagnoses. In all studies reporting treatment history, participants had not responded to prior interventions targeting EBSA.

#### 3.2.2 Program characteristics

Thirteen programs were identified. Program structures comprised inpatient hospital programs (n=6), alternative education programs (n=3), day hospital programs (n=2) and outpatient clinics (n=2). Most programs (n=11) were staffed by multidisciplinary teams comprising of mental health and educational professionals. The remainder (n=2) were staffed solely by mental health clinicians. Most programs (n=12) involved participation four or more days per week. Among programs with extractable duration data (n=8), treatment length ranged from 8 to 50 weeks (median= 15 weeks).

The most common theoretical frameworks were cognitive–behavioural (n=3), behavioural (n=3), and ecological (n=3). Other frameworks included systemic (n=2), schema (n=1), and dialectical-behavioural (n=1). Most programs (n=11) combined psychotherapy and non-psychotherapy components. Psychotherapy components included individual therapy (n=11), group therapy (n=9), parent therapy (n=8), family therapy (n-6), parenting group therapy (n=2), and multi-family therapy (n=2). Non psychotherapy components included case management (n-12), supported classroom provision (n=10), pharmacotherapy (n=4), and creative arts therapies (n-3). Most programs (n=11) integrated young people into the treatment program before supporting transition to their next educational setting.

#### 3.2.3 Reporting characteristics

Reporting on referral pathways, eligibility criteria and participant selection was variable. Among samples specifying referral sources (n=8), pathways included outpatient mental health services (n=5), education systems (n=3), and direct research clinic referrals (n=1).

Among samples specifying eligibility criteria (n=11), the most common criteria were presence of a psychiatric disorder (n=8), non-attendance rate (n=7), age (n=7) and fulfilment of EBSA criteria (n=7). Non-attendance thresholds were typically defined as complete or near complete absence over the preceding two to six weeks. Diagnostic requirements most commonly specified anxiety and/or depressive disorders (n=5). The most common eligibility configuration combined a psychiatric diagnosis with a non-attendance threshold. Common exclusion criteria (n=8) included intellectual disability (n=8), psychotic disorders (n=6), autism spectrum disorder (n=6), and conduct disorder (n=5).

Only five studies reported participant selection data sufficiently to determine the proportion entering intensive treatment relative to those referred or assessed for eligibility.

Reporting on EBSA characteristics was heterogeneous (see **Table 3**; **Supplementary Appendix**). Severity of non-attendance (15/17) and psychiatric comorbidity (15/17) were frequently reported. Chronicity of attendance problems (11/17) and prior treatment history (7/17) were less consistently reported. Theoretical orientation was either explicitly reported or could be classified from program descriptions in all programs.

#### 3.2.4 Outcome domains and longitudinal follow-up

Outcome reporting was heterogeneous across studies, with substantial variation in both the domains assessed and the duration of follow-up. Of studies reporting post-intervention outcomes (14/19), most reported school attendance (n = 11), typically as a proportion of days attended, or categorical attendance improvement. Other outcome domains included symptomatology (n = 8), global functioning (n = 7), educational setting (n = 1), school refusal behaviour (n = 1), and quality of life (n = 1). Longitudinal post-intervention outcomes were reported by over half the studies (n = 8), ranging from three months to three years post-intervention. Outcome findings are interpreted within program types in Section 3.3.

### 3.3 Program types

For analytic clarity, the 13 identified programs were grouped according to their primary treatment setting and delivery structure, yielding four program types: intensive outpatient therapy (n = 2), alternative education programs (n = 3), day hospital programs (n = 2), and inpatient programs (n = 6). These program types differed in referral pathways, eligibility thresholds, structural intensity, and their positioning within mental health and education systems.

#### 3.3.1 Intensive outpatient therapy

Four publications described two intensive outpatient programs delivered in clinic-based settings in the USA as first line treatments for young people with school refusal and comorbid psychiatric disorders. A brief, daily cognitive–behavioural therapy (CBT) program was reported across two publications, the first describing an initial pilot case series (n=4) (36) and the second a larger replication sample (n=25) (29). The program comprised approximately 30 hours of therapy delivered over three weeks. Treatment was guided by functional analysis of maintaining factors and incorporated parent work and structured liaison with the school. Referrals and program funding were provided through local school districts, reflecting the close involvement of the education system in program delivery. In the replication sample, 84% of participants completed treatment and 60% achieved satisfactory school attendance (defined as >90% attendance) in the post-treatment phase.

A pilot feasibility study of dialectical behaviour therapy adapted for SR (DBT-SR) was reported across two publications (24, 25). The 16-week program included weekly individual and parent sessions, daily web-based coaching, and multi-family skills groups incorporating school-related exposures. Of four families enrolled, two discontinued early; one of the two completers achieved full school return.

#### 3.3.2 Alternative education programs

Three publications from Australia (32), the Netherlands (23), and England (28) described time-limited alternative education programs designed for young people with EBSA. These programs were positioned within the education sector rather than mental health services. Referral pathways were commonly through schools or educational authorities and were typically contingent on meeting EBSA criteria rather than the presence of a specific psychiatric diagnosis. The Australian In2School program (32) was a partial exception, receiving referrals solely through a co-located tertiary mental health service. Samples were heterogenous; notably the Dutch The Link program included a high proportion of young people with autism spectrum disorder.

Functional gains were pursued through shaping classroom ecology and school climate. Core features included small class sizes, predictable routines, individualised learning plans, graded entry processes, and structured group activities aimed at rebuilding confidence and peer connection. Where individual therapeutic input was provided, this was typically informal and delivered on an as-needed basis, often in parallel with external mental health care rather than integrated within the program itself. All programs had set intervention durations; however extended admissions were a common theme (23, 28). For instance, in The Link program, mean duration was 12 months, double the intended duration.

Only the *In2School* program reported longitudinal attendance outcomes. Five of seven students maintained adequate attendance (>70%) at six-month follow-up. *The Link* program reported on educational settings pre- and post-intervention. While 70% of students had been enrolled in mainstream settings pre-intervention, 79% had transitioned to specialised educational settings post-program. *The Link* program also analysed whether ASD status influenced length of admission, finding no difference between adolescents with ASD and those with other primary diagnoses.

#### 3.3.3 Day hospital programs

Three studies described two day hospital programs in France (26, 27) and Germany (30). Day hospital programs integrated multidisciplinary mental health treatment with embedded educational provision delivered through a hospital-based classroom for young people with chronic and often complete EBSA associated with anxiety or depressive disorders. Young people attended four to five days per week, participating in a daily timetable that paired structured psychological therapies with educational activities. Reported program duration ranged from 15 to 30 weeks. Reintegration to the home school was introduced in a graded manner during the latter phase of treatment, with therapeutic and educational staff coordinating transition planning. Across studies, high rates of school return were reported by program completion or follow-up. In the retrospective French and German cohorts, 88–95% of young people achieved regular school attendance by completion or follow-up. In a subsequent multicentre prospective phase II study of the French program, 53% achieved ≥80% school attendance at 6 months, with improvements in anxiety symptoms and global functioning maintained at 12-month follow-up. All studies used uncontrolled designs, and reporting on participant selection and attrition was limited.

#### 3.3.4 Hospital inpatient programs

Eight publications described five hospital inpatient programs based in Germany (33, 37–40), France (22), Japan (31) and Turkey (34). Three programs involved specialised units, two designed specifically for EBSA (31, 33, 37–40) and one for addiction, including internet gaming disorders (22). The remainder described inpatient care delivered within general psychiatric units (31, 34).

In the specialised programs, a classroom was embedded within the treatment setting and young people participated in daily educational activities alongside therapeutic and self-care tasks. Across programs, treatment commonly targeted sleep-wake regulation, self-care, and behavioural activation (31, 34). Psychotherapy models — cognitive behavioural therapy (40), schema therapy (33), and multidimensional family therapy (22) — were treatment components delivered to the young person and parent, as well as organising frameworks for unit-wide practice across clinical and educational staff (33). Mean intervention durations ranged from 8–12 weeks. Outcomes were most comprehensively reported in the German inpatient CBT program (39, 40). Across two samples, 82 – 86% of young people achieved satisfactory school attendance (> 90%) two months post-intervention. Longitudinal outcomes at 9 months post-intervention were collected in one sample, with 64% maintaining satisfactory attendance.

In contrast, treatment programs delivered in general psychiatric settings relied predominantly on behavioural therapy, often with limited educational components of treatment. A Japanese program delivered sleep-wake modification therapy to young people with delayed sleep phase disorder and comorbid EBSA (31). In a prospective cohort (n=22), approximately half the young people demonstrated satisfactory school attendance (defined as > 70%) at 3-month follow-up. A Turkish program described graded exposure therapy for young people in whom outpatient care had been ineffective. A case series (n=2) reported satisfactory school return at 6-month follow up (34).

### 3.4 Therapeutic levers

Comparative analysis across the four program types identified several recurring processes that were consolidated into higher-order therapeutic levers. Educational scaffolding (n = 9), therapeutic peer milieu (n = 6), health and education systems coordination (n = 6), high intensity graded exposure (n = 6) and transitional space (n = 5) were the most frequently identified therapeutic levers. Other processes—including self-related processes (n = 3), parental accommodation (n = 3), and family reorganisation (n = 2)—were less consistently specified.

#### 3.4.1 Educational scaffolding

Educational scaffolding—defined here as the structured and ongoing calibration of educational demands to a young person’s capacity—operated as a core therapeutic lever in all programs with embedded educational provisions. By embedding educational demands within the intervention itself, programs were able to shape, pace, and monitor participation directly, rather than relying on external implementation by home schools or families.

Scaffolding operated across multiple levels. Environmentally, programs employed small student–staff ratios, additional support personnel, low-stimulation settings, tailored academic expectations, shortened hours, and contingency plans for distress (23, 26, 30, 32). Relationally, scaffolding was enacted through consistent staff who prioritised trust-building and engagement, often supported by training in mental health or behaviour support (30, 32, 33). Procedurally, scaffolding involved ongoing tailoring informed by baseline assessment and adjusted over time in response to engagement, distress, and functional capacity (23, 26, 30).

#### 3.4.2 Therapeutic peer milieu

The peer milieu, conceptualised here as the peer component of the therapeutic environment, emerged as a further core lever in group-based programs (23, 26, 30, 32, 33, 35). Across studies, young people with chronic or severe EBSA were commonly described as experiencing social withdrawal, loneliness, and shame (33, 35). Entry into a peer context comprised of young people with similar histories appeared to provide a corrective social experience, reducing perceived difference and reframing avoidance as an understandable coping strategy rather than a failure and source of stigma (35).

Within this context, programs created opportunities for peer-to-peer learning. In some programs, peers were actively incorporated into program tasks; for example, in one program during initial return-to-school visits, the young person was accompanied by a peer, a strategy that provided peer support for the integrating student and modelling for the observing peer (26).

Importantly, these peer effects were not confined to formal group therapy. Several programs described peer processes operating through everyday shared routines, classroom participation, and informal interactions (23, 32, 33). In these contexts, peer presence functioned as an ongoing social regulator of participation, with norms around attendance and engagement sustained laterally within the group rather than solely enforced by staff (33). A smaller subset of programs promoted parent-to-parent peer processes through multi-family and/or parent group therapy (22, 24, 30, 32).

#### 3.4.3 Health—education systems coordination

Coordination between mental health and educational systems functioned as a lever across programs. Internally, coordination was achieved through close alignment of health and education staff within the program itself. Examples included educational staff receiving training in therapeutic models (33), mental health clinicians embedded within classroom settings (32), clinical teams actively shaping educational delivery (23), and joint health–education meetings with families to support shared formulation and planning (23, 32). This alignment supported consistent messaging, shared decision-making, and alignment of therapeutic and educational goals.

Externally, systems coordination involved deliberate efforts to mobilise and prepare support within the young person’s home school. Programs described proactive engagement with schools prior to reintegration, identification of key support figures (e.g., designated mentors or coordinators), and the establishment of agreed expectations and support structures before the young person’s return (28, 30, 32).

#### 3.4.4 High intensity graded exposure, and strategies to sustain participation

High intensity graded exposure was a core feature of several programs. Intensity was generated structurally through program design, with interventions involving daily or near-daily contact over several weeks, thereby compressing exposure cycles that might otherwise unfold slowly in standard outpatient care. This high frequency enabled repeated, closely spaced exposures with rapid calibration of exposure difficulty, reducing opportunities for loss of treatment gains between sessions.

In addition to structural intensity, programs used several strategies to sustain participation. Program entry procedures often operated as an early engagement lever, with eligibility contingent on young people and families actively endorsing the goal of return-to-school and accepting the program as an appropriate pathway (23, 26, 30, 32). A small number of programs (n = 3) reinforced participation through explicit contingencies, ranging from dismissal following repeated non-attendance in intensive outpatient models (29, 36) to inpatient approaches that linked progression or discharge to active engagement (34). Other programs reduced the viability of non-participation through structural supports that enabled daily attendance, including program-provided transport and stepwise build-up of attendance demands (30). A broader subset of programs incorporated formal therapy components to support persistence—most notably structured motivation-and-goal modules (38) and parent-focused validation/coaching strategies delivered at key avoidance points (24).

#### 3.4.5 Transitional space

In several programs, the intervention site functioned as a transitional space: a temporary, intermediate setting that was neither home nor mainstream school and was explicitly oriented toward reintegration rather than permanence.

At a practical level, several programs operationalised this transitional function through graded entry and exit, with young people supported to move incrementally between settings. Examples included stepwise increases in program attendance hours, accompanied visits to mainstream classrooms, and staff-supported graded reintegration to the home school (26, 30, 34). These approaches positioned the program as a training ground for reintegration, allowing skills, routines, and tolerances developed within the program to be tested and transferred *in vivo*.

The transitional space also carried psychological and relational significance. For young people, participation in a setting that was neither home nor their previous school offered relief from the accumulated meanings, failures, and expectations associated with earlier schooling. In qualitative accounts, this was described as a corrective experience that supported processes of self-reorientation, with some young people beginning to shift their identities away from illness or failure (35). Importantly, this occurred not in the absence of educational demands, but within a context where those demands were recalibrated and reintroduced under conditions of support.

### 3.5 Implementation challenges

Across the 13 programs, engagement—encompassing both the young person and parents—was the most frequently identified implementation challenge (n = 8). Other recurring implementation challenges included progression through the program (n = 4) and resourcing constraints (n = 4). Maintenance of gains following intervention (n=3) and inter-system challenges (n=1) were infrequently identified.

#### 3.5.1 Engagement

Sustaining engagement—both of young people and their parents—was reported as a challenge across several programs. Of programs reporting attrition, between 10 – 50% of participants dropped out (24, 27, 38). Two patterns of disengagement were described: early drop out in the first days of treatment (24, 38), and gradual disengagement over several weeks (24). Parental factors were commonly associated with disengagement. Limited parental engagement was identified as a barrier in some programs (29, 30, 36). Similarly, parental anxiety or ambivalence about their child's school re-integration was observed to undermine the young person’s commitment to change (28).

Several strategies were employed to sustain engagement. These strategies generally sought either to increase commitment to treatment at entry or to reduce practical barriers to ongoing participation. Most programs applied selection thresholds at entry, requiring commitment to return-to-school goals from the young person and family (26, 32, 38). A few programs employed explicit contingencies. An American outpatient program made continuation of treatment contingent on active participation (29, 36). A Turkish inpatient unit made progression to discharge contingent on engagement in school exposure (34). More programs opted to reduce barriers to program engagement through structural supports. A German day hospital program provided daily transport of young people from home to the program site (30). An intensive outpatient DBT program provided daily web-based coaching in the mornings to support parents in promoting the young person’s school attendance. In instances when young people disengaged from the DBT program, treatment could be continued indirectly via parent therapy (24).

#### 3.5.2 Progression

Progression through treatment emerged as a recurring implementation challenge, reflected in the wide variation in treatment duration both within and between programs. Amongst programs using short, fixed durations, treatment was structured around predefined timeframes with an emphasis on early reintegration. In qualitative research on a six-week Short Stay School in the UK, an emergent theme was of young people and carers experiencing pressure to progress through the program. In longer, or more flexible programs, extended admissions and delayed reintegration were described as challenges. In the Dutch *The Link* program, there was a wide margin between the expected program duration (6–12 months) and actual treatment times, with some cases extending beyond two years. Determining appropriate duration of treatment, readiness for integration, and ways to improve program efficiency were reported as challenges.

#### 3.5.3 Resourcing

Several programs highlighted the resource demands of intensive delivery models. Intensive outpatient CBT required substantial clinician time and in some cases was only feasible within specific funding arrangements, such as support from school districts (29, 36). Care coordination across health and education systems was identified as another strain on resources, with challenges including unclear referral pathways, duplication of work, and the time investment needed to maintain joint working (32). Additional structural supports to facilitate engagement—such as transport, outreach, and parent coaching—further extended resource requirements (24).

Conversely, some programs were explicitly developed in response to perceived limitations of existing service models. The German schema therapy day program came into being in response to poor outcomes from conventional outpatient care; a joint health-education intensive program was identified as a more efficient use of resources over time, given the persistence of school attendance difficulties following standard outpatient or inpatient treatment and the associated long-term educational and social costs (33).

## 4 Discussion

EBSA is a major multi-system challenge at the intersection of mental health and education, requiring coordinated and stratified responses across both sectors (42). Addressing EBSA is less about enforcing attendance and more about facilitating re-engagement in learning and socialising, as a means of restoring healthy developmental trajectories in an at-risk population, in preparation for the transition to adulthood (43). For many young people this can be achieved through school-based and outpatient interventions. However, a subset experience persistent and severe attendance difficulties despite standard mental health and educational supports, creating the need for more intensive forms of intervention. Intensive interventions have long been recognised as a treatment option for young people with chronic and severe forms of EBSA (6, 12), yet the empirical literature describing these interventions remains fragmented. To address this gap, we conducted a scoping review to map and conceptualise participant characteristics, intervention structure and delivery, recurring therapeutic levers and implementation challenges, and reporting completeness across studies of intensive EBSA interventions.

### 4.1 Intensive EBSA interventions: a field early in development

The identified literature on intensive EBSA interventions reflects a field still in the early stages of development. Most reports comprised single-arm observational studies, case reports, and program descriptions rather than controlled evaluations. Reporting across studies was variable and often incomplete, with inconsistencies in the description of referral pathways, participant selection, intervention components, and outcome measurement. Theoretical foundations and classification frameworks were heterogeneous, reflecting the diverse service contexts in which these programs have evolved. As a result, the literature has primarily focused on describing program models and reporting local service outcomes, with limited attention to mechanisms of change or comparative effectiveness.

### 4.2 Treatment delivery: familiar components, different conditions

Within this heterogeneous and early-stage literature, several consistent patterns can be identified in how intensive EBSA interventions are structured and delivered. Notably, the components commonly delivered in intensive programs—structured psychotherapy, parent support, graded exposure, educational supports, and school liaison—resemble evidence-based Tier 3 interventions (10, 44). What was different, however, were the structural conditions under which treatment was delivered. Tier 3 interventions are commonly delivered in individual or family-based formats in clinic settings and rely on the family and external school to enact change. In contrast, most of the intensive programs examined utilised site-based and group-based formats. The site typically included a therapeutic classroom supported by a multidisciplinary team comprising health and education staff. The group-based delivery incorporated a therapeutic peer milieu. It is plausible, then, that intensive programs may succeed in circumstances where Tier 3 interventions have not—not because they introduce novel therapeutic techniques, but because they create an environment in which evidence-based treatments can be implemented more consistently.

### 4.3 Therapeutic levers: how structure enables change

The therapeutic levers identified across program types suggest several plausible mechanisms through which intensive interventions may facilitate engagement and recovery. The on-site classroom allows for educational scaffolding of emotional, social, and academic tasks, calibrated to the young person’s capacity. The peer milieu may reduce isolation and normalise program participation, providing a powerful antidote to avoidance as a default coping strategy. Integrated staffing may reduce the risk of fragmentation between health and education systems, both within the program and across external stakeholders. Finally, the transitional space created by the program site provides a bridge between home and school. The time-limited quality of this transitional space may create a sense of urgency that mobilises resources for change. Together, these structural conditions may reduce barriers and amplify facilitators of engagement, shifting the balance sufficiently to allow evidence-based treatments to be delivered more reliably than in Tier 3 interventions. These findings are consistent with broader complex-intervention frameworks, in which change emerges not from a single therapeutic component but from the interaction of multiple environmental, relational, and organisational conditions (19).

Notably, these levers converge with stakeholder-derived ‘signposts’ for EBSA intervention, particularly the emphasis on lowering initial barriers to engagement through the provision of safe, structured, and relationally supportive environments (45). Taken together, these observations point to a set of structural ‘therapeutic levers’ that warrant more explicit specification and empirical testing in future research, including examination of their relative contribution to outcomes and the conditions under which they are most effective.

It is also worth reflecting on the levers that were less consistently identified across programs. There is an extensive literature on the role of family systems in EBSA (46) — one might expect intensive interventions to consistently leverage family reorganisation to mobilise change. Whilst multiple programs targeted reduction in parental accommodation (24, 29, 36) and family system boundaries (22, 33), family reorganisation did not emerge as a consistently specified cross-program therapeutic lever. This may reflect their embedding within broader program delivery or their secondary role; further work is needed to clarify the role and specification of family-level mechanisms within intensive intervention models.

### 4.4 Implementation challenges: tensions in program delivery

The implementation challenges identified across programs point to several underlying tensions that remain under-explored in the literature. The first concerns engagement and reflects a tension between accessibility and readiness for participation. Intensive programs must balance lowering the barriers for entry into treatment against the need to select young people who are able to engage with intensive support. Strategies that emphasise readiness at entry may limit access for the most impaired, whereas more inclusive approaches may be associated with higher attrition. This tension is compounded by the ambivalence that commonly characterises severe EBSA, in which young people and families may simultaneously express a desire for change and apprehension at the uncertainty it entails. Readiness for engagement is therefore not a stable or easily defined construct, but one that fluctuates over time and across contexts. A better understanding of how programs negotiate this tension is constrained by sparse reporting on referral flow, participant selection and attrition, and limited qualitative research on the barriers and facilitators to engagement.

A second challenge concerns progression through treatment and reflects a tension between containment and reintegration. Programs must balance the provision of a safe and supportive environment that facilitates initial engagement with the need to promote timely reintegration. Short, time-limited models may generate momentum toward reintegration but risk premature transition, whereas longer or more flexible models may support stabilisation at the cost of delayed progression and increased resource use. As with engagement, understanding how programs navigate this tension is limited by sparse reporting on program duration, criteria for progression, and factors associated with delayed reintegration.

A third challenge concerns resourcing and reflects a tension between intensity and scalability. Intensive programs concentrate resources to create conditions that generate change. However, this concentration of resources raises questions about scalability, equitable access, and sustainability. On the other hand, the limited effectiveness of lower-intensity interventions for a sub-group of young people with EBSA raises the possibility that repeated or prolonged Tier 3 care may itself represent an inefficient use of resources. Indeed, it may be the case that intensive interventions are justified not only on clinical grounds but also as a means of reducing longer-term educational, social, and service burden. Despite this, the balance between short-term investment and longer-term efficiency remains largely unexamined. Few studies incorporate economic or service-use outcomes, and there is limited evidence to guide decisions about when intensive intervention should be deployed or how resources should be optimally allocated across tiers of care.

### 4.5 Tiered care: the place of intensive interventions

Our findings have important implications for how intensive EBSA interventions are conceptualised within multi-tiered frameworks for school attendance problems. The program-type analysis suggested two broad ways in which intensive interventions may address barriers to engagement in treatment: i) escalation of treatment intensity and ii) environmental restructuring to enable engagement with treatment and schooling.

Interventions such as intensive outpatient therapy primarily increase the intensity or frequency with which evidence-based treatments are delivered. These models do not substantially modify the treatment environment and, within the studies identified in this review, were evaluated exclusively as first-line interventions for EBSA. One interpretation of these findings is that intensive outpatient therapy may be better conceptualised as a high-intensity extension of Tier 3 care (“Tier 3+”) rather than a Tier 4 intervention. The available evidence suggests that the effectiveness of intensive outpatient therapy is contingent on strong family and home school capacity to support exposure work. Determining which young people benefit from intensive outpatient therapy over less intensive evidence-based outpatient treatments is an important topic for future research.

In contrast to intensive outpatient therapy, the remaining program types—alternative education programs, day hospital programs, and inpatient programs—modify the environmental context in which treatment occurs, creating settings in which evidence-based interventions can be delivered and engagement in school and other daily activities re-established. These program types align more closely with Kearney’s description of Tier 4 interventions as ‘specialised programs’. The available evidence suggests that the majority of young people accessing these programs make gains in school attendance and global functioning. It remains unclear, however, whether the young persons enrolled in such programs constitute the cohort of young people “with such severe and chronic attendance problems that even extensive Tier 3 efforts may be inadequate” that Kearney originally described as the target population of Tier 4 interventions. Few studies sufficiently described EBSA severity or documented non-response to evidence-based Tier 3 interventions to make meaningful conclusions about the effectiveness of interventions for *severe* EBSA.

Further, most of the intensive programs evaluated in this review were described as stand-alone services rather than components of staged care pathways, making it unclear how intensive interventions fit within a tiered system of supports. It therefore remains unclear whether participant selection in intensive programs is primarily determined by clinical severity or local service configuration. Clarifying this distinction will be critical for helping professionals and services determine which young people are most likely to benefit from intensive EBSA interventions, when such interventions should be deployed, how they should be evaluated, and how they can be integrated within tiered systems of care.

## 5 Research priorities for intensive interventions for EBSA

The review identified several recurring evidence gaps relating to participant selection, participant characterisation, program specification, and outcome measurement. These gaps provide a preliminary agenda for future research on intensive interventions for EBSA.

### 5.1 Staging & severity

A major gap identified in this review was the limited characterisation of severity among young people receiving intensive interventions. Although many samples were described as chronic or severe, reporting of non-attendance severity, chronicity, psychiatric comorbidity, and prior treatment history was often incomplete. As a result, it remains difficult to determine whether intensive programs are serving treatment-refractory populations, young people with the most severe forms of EBSA, or populations defined primarily by local service configuration. Greater precision in describing severity would improve interpretation and comparison across studies.

Future research would benefit from more consistent reporting of: (i) proportion of non-attendance; (ii) duration of non-attendance below 50%; (iii) psychiatric and neurodevelopmental comorbidity; and (iv) prior Tier-3 treatment exposure and response.

Future research may also benefit from exploring staging approaches capable of distinguishing different levels of EBSA severity, particularly among young people with chronic or near-complete non-attendance. Improved differentiation of severity may help clarify which young people are most likely to benefit from intensive interventions and facilitate comparison across studies and service models.

### 5.2 Report participant selection, representativeness, and treatment flow

A major gap identified in the review was limited reporting of referral pathways, participant selection, and treatment flow. Future research would benefit from reporting referral and selection flow using clear denominators: i) the number of young people assessed for eligibility, ii) the number eligible, iii) the number offered an intensive program, iv) the number who commenced, and v) the number who completed. Reasons for non-entry and attrition should be described explicitly (e.g., refusal, contraindications, competing placements, risk, family barriers, logistical barriers), alongside key baseline differences between those who enter and those who do not.

This reporting is essential to distinguish program effectiveness from selection effects and to address a central tension in intensive EBSA interventions: intensive programs may require a minimum level of readiness to participate, yet they are also intended to reduce barriers to access and engagement for young people who have not been able to engage in less intensive care. Transparent selection and flow reporting provides the foundation for subsequent work examining engagement mechanisms and the treatment gap.

### 5.3 Specify program structure and core components

The review identified considerable heterogeneity and incomplete reporting of program structure. Greater specification of structural elements would improve replication, comparison, and future investigation of how the identified therapeutic levers generate change. Future studies would benefit from clearly documenting: (i) program setting; (ii) duration and weekly intensity (hours per week); (iii) composition and roles of multidisciplinary staff; (iv) presence and structure of an on-site classroom; (v) group-based versus individual modalities; (vi) parent involvement; and (vii) processes for reintegration or transition back to mainstream education. Without detailed description of program architecture, it is difficult to determine which components are necessary, which are adjunctive, and which may be scalable across settings.

### 5.4 Strengthen study design using complex-intervention approaches

The predominance of descriptive and uncontrolled studies identified in this review highlights the need for stronger evaluative designs. Because these programs are complex, multi-component interventions embedded within health and education systems, conventional individually randomised controlled trials may not always be feasible.

Nevertheless, a range of approaches used in complex intervention research may help address current evidence gaps. These include pragmatic-controlled trials, comparative cohort studies, cluster designs, quasi-experimental approaches, and evaluations embedded within stepped-care pathways.

Such approaches may allow investigation of the incremental contribution of intensive interventions beyond standard outpatient care while preserving real-world service delivery. Future research may also benefit from comparing programs that differ in timing of referral, duration, intensity, or structural features, and from examining whether outcomes vary according to severity, psychiatric comorbidity, or broader service and systems context.

### 5.5 Standardise outcome measurement and broaden outcome domains

The review identified substantial heterogeneity in outcome domains, measurement approaches and follow-up periods. Attendance may be the primary behavioural outcome in EBSA research, but it provides only a partial account of program impact. Consistent with recommendations by Heyne and colleagues (47), future research would benefit from measuring school attendance alongside key clinical and functional constructs, including emotional symptoms (particularly anxiety and depression), behavioural functioning, global functioning, and broader indicators of wellbeing and family adjustment. The primary limitation of the existing intensive intervention literature is not the absence of such outcomes, but substantial variability in which constructs are measured, how they are operationalised, and when they are assessed, which limits comparison and synthesis across studies.

Greater consistency in the domains measured, the instruments used, the timing of measurement, and the informants providing data would improve interpretability and comparability across studies. Attendance should ideally be derived from school-record data and reported as the proportion of scheduled time attended over a defined period. Where feasible, additional contextual indicators (e.g., partial-day attendance, late arrival, or reliance on escorted entry) may help distinguish supported attendance from independent reintegration.

Future research would also benefit from capturing the context of educational reintegration. Attendance outcomes should therefore specify not only how much school is attended, but where attendance occurs—such as return to mainstream schooling versus participation in supported or alternative educational settings—and the stability of that placement over time.

Finally, the review identified very limited reporting of economic and service-use outcomes despite the cross-sector and resource-intensive nature of intensive interventions. Future studies would benefit from incorporating such outcomes. These may include the financial and opportunity costs experienced by families during prolonged non-attendance, as well as changes in family burden, service utilisation, and educational placement following intervention.

### 5.6 Investigate mechanisms of change in intensive programs

The review identified several recurring therapeutic levers across intensive programs; no studies, however, sought to test the specific mechanisms that generate change. Future research would benefit from moving beyond program description toward empirical investigation of mechanisms of change. Priority areas include understanding how intensive program environments reduce barriers to engagement for young people who have not responded to outpatient care; identifying classroom practices that support graded academic and social re-engagement; and examining family processes that support change during the program and maintain gains following treatment. Such work may help determine which structural elements are most important for successful intervention and which may be adapted across service settings.

## 6 Limitations

First, as a scoping review, the objective was to map and synthesise the range of intensive EBSA programs described in the literature rather than to formally evaluate study quality or estimate treatment effects. Consistent with scoping review methodology, we did not undertake a formal risk-of-bias appraisal. Consequently, the review does not provide conclusions regarding the comparative effectiveness of intensive programs.

Second, substantial heterogeneity and incomplete reporting across studies constrained data extraction and synthesis, limited direct comparison across programs, and reduced confidence in conclusions regarding participant selection and the indications for intensive intervention.

Third, the search strategy was limited to peer-reviewed publications and dissertations indexed in major academic databases. Intensive programs for EBSA are often embedded within local service systems and may be described primarily in service reports, internal program evaluations, or other forms of grey literature that are not indexed in academic databases. It is therefore likely that additional programs operating in this space were not captured. The findings should therefore be interpreted as reflecting the published evidence base, rather than the full range of programs currently operating in practice. In addition, a small number of non-English publications were screened using Google Translate. Although this approach facilitated inclusion of non-English studies, translation inaccuracies may have affected interpretation of intervention components, therapeutic levers and implementation challenges.

Fourth, in the absence of a consensus definition, we operationalised ‘intensive’ as time-limited programs designed to treat EBSA. Whilst this approach enabled comparison across programs, it excluded other relevant service models. For example, some specialist education settings support young people with EBSA on an open-ended basis, while some intensive mental health programs (e.g., day hospital or inpatient services) treat substantial numbers of young people with EBSA without describing EBSA-specific interventions. The review therefore reflects programs that explicitly conceptualised EBSA as a treatment target rather than all intensive services that may support this population.

Fifth, the conceptual framing of intensive programs reflects the perspectives through which the literature was interpreted. Although the review drew on frameworks spanning health and education sectors, much of the published literature is situated within mental health services. As a result, the synthesis may place greater emphasis on therapeutic and service-delivery structures than on educational practice or pedagogy.

Despite these limitations, the review provides a systematic synthesis of the emerging literature on intensive EBSA programs and offers a framework for future research examining how such programs may be positioned within multi-tiered intervention systems.

## 7 Conclusions

Intensive interventions for EBSA represent a diverse but conceptually coherent set of approaches comprising intensive outpatient, alternative education, day hospital, and inpatient program models. Across most program types, the defining feature of these interventions is not the use of novel therapeutic techniques, but the structural conditions under which treatment is delivered. By embedding care within site-based, group-based, and multidisciplinary settings, intensive programs appear to reduce barriers to engagement and enable more consistent implementation of evidence-based treatment components.

As a scoping review, this study was designed to map and synthesise the available literature rather than evaluate comparative effectiveness. Substantial heterogeneity and incomplete reporting across studies constrained synthesis and limited direct comparison across programs. Future research would benefit from clearer specification of program structure, improved characterisation of participant populations, and empirical investigation of how the identified therapeutic levers generate change. Such work may support development of a stronger evidence base to guide the role of intensive interventions within multi-tiered frameworks for school attendance problems.

## Data Availability

The original contributions presented in the study are included in the article/**Supplementary Material**. Further inquiries can be directed to the corresponding author.
