# Supplementary material for: Intensive interventions for emotionally based school avoidance: a scoping review of program structures and therapeutic levers
Source: Front Psychiatry. 2026 Jul 31;17:1851117. doi: 10.3389/fpsyt.2026.1851117 (PMC13473371; doi:10.3389/fpsyt.2026.1851117)
Supplement: Supplementary file 1 [file DataSheet1.docx]

Appendix 1. Search Strategy

**Electronic Databases**

The following databases were searched from 1 January 2000 to 24 May 2025:

- MEDLINE (Ovid)
- Embase (Ovid)
- PsycINFO (Ovid)
- ERIC (Ovid)
- ProQuest Dissertations & Theses Global

No language restrictions were applied during database searching.

**Search Concepts**

Search strategies combined terms relating to:

1. School refusal and related attendance problems
2. Intensive intervention settings and programmes

The school refusal concept included terms such as:

("school refus*" OR "school phob*" OR "emotion* based school avoid*" OR "emotion* based school absen*" OR "emotionally based school avoid*" OR "emotionally based school absen*" OR "school avoidance" OR "school non-attend*" OR "school disengage*" OR "school withdrawal" OR "school dropout")

The intensive intervention concept included terms such as:

("intensive" OR "day program" OR "day patient" OR "partial hospital*" OR "inpatient" OR "residential treatment" OR "hospitali*" OR "alternative education program*" OR "alternative provision" OR "flexible learning" OR "therapeutic program" OR "specialist education setting")

Concepts were combined using the Boolean operator AND. Search syntax was adapted for individual databases.

**Example Search Strategy (MEDLINE/Ovid)**

(("school refus*" OR "school phob*" OR "emotion* based school avoid*" OR "emotion* based school absen*" OR "emotionally based school avoid*" OR "emotionally based school absen*" OR "school avoidance" OR "school non-attend*" OR "school disengage*" OR "school withdrawal" OR "school dropout"))

AND

(("intensive" OR "day program" OR "day patient" OR "partial hospital*" OR "inpatient" OR "residential treatment" OR "hospitali*" OR "alternative education program*" OR "alternative provision" OR "flexible learning" OR "therapeutic program" OR "specialist education setting"))

**Grey Literature**

Grey literature was searched through ProQuest Dissertations & Theses Global using adapted search terms relating to school refusal and intensive intervention settings.

ProQuest Dissertations & Theses Global was selected as the primary source of grey literature due to its extensive coverage of doctoral and master's theses. However, submission is not mandatory in many countries and some relevant theses may be available only through institutional repositories or national databases.

**Supplementary Searching**

To identify potentially relevant studies not captured through database searching, supplementary searches were conducted in Google Scholar (7 June 2025) using combinations of terms relating to school refusal and intensive intervention settings (e.g., "partial hospitalization", "day program", "alternative education", "school refusal", "school avoidance", and "school absenteeism").

Reference lists of included studies and relevant review articles were also examined for additional eligible publications.
